# Body fat and muscle were associated with metabolically unhealthy phenotypes in normal weight and overweight/obesity in Yi people: A cross-sectional study in Southwest China

**DOI:** 10.3389/fpubh.2022.1020457

**Published:** 2022-10-06

**Authors:** Ye Wang, Li Pan, Shaoping Wan, Wuli Yihuo, Fang Yang, Zheng Li, Zhengping Yong, Guangliang Shan

**Affiliations:** ^1^School of Population Medicine and Public Health, Chinese Academy of Medical Sciences and Peking Union Medical College, Beijing, China; ^2^Department of Epidemiology and Statistics, Institute of Basic Medical Sciences, Chinese Academy of Medical Sciences and Peking Union Medical College, Beijing, China; ^3^School of Medicine, Sichuan Cancer Center, Sichuan Cancer Hospital & Institute, University of Electronic Science and Technology of China, Chengdu, China; ^4^Puge Center for Disease Control and Prevention, Liangshan, China; ^5^Xichang Center for Disease Control and Prevention, Liangshan, China; ^6^Sichuan Academy of Medical Sciences and Sichuan Provincial People's Hospital, Chengdu, China

**Keywords:** body composition, metabolically healthy overweight/obesity, Yi people, bioelectrical impedance analysis, muscle-to-fat ratio

## Abstract

This study aimed to determine the association between the absolute mass, distribution, and relative ratio of body fat and muscle with the metabolically unhealthy (MU) phenotypes in normal weight and overweight/obesity in Yi people in China. The cross-sectional data from the Yi Migrants Study was used, which included 3,053 Yi people aged 20–80 years from the rural and urban sets. Participants were classified according to body mass index and metabolic status. Body composition including body fat percentage (BFP), fat mass index (FMI), visceral fat grade (VFG), muscle mass index (MMI), and muscle/fat ratio (M/F) were measured by bioelectrical impedance analysis. Restricted cubic spline and logistics regression models were used to test the associations between body composition parameters with MU phenotypes. Receiver-operating characteristic curves (ROC) were used to analyze the predictive value of MU phenotypes. Among the normal weight and overweight/obesity, 26.31% (497/1,889) and 52.15% (607/1,164) were metabolically unhealthy. Stratified by BMI, covariance analysis showed higher body fat (BFP, FMI, and VFG) and MMI in MU participants than in healthy participants. BFP, FMI, VFG, and MMI were positively associated with MU phenotypes both in normal weight and overweight/obesity after adjustment. M/F was significantly lower than MU participants and was negatively associated with MU phenotypes. BFP, FMI, VFG, and M/F could better predict MU phenotypes than BMI. We concluded that BFP, FMI, and VFG were positively associated with MU phenotypes, while M/F was negatively associated with MU phenotypes across the BMI categories in Yi people. Body fat and muscle measurement could be a valuable approach for obesity management.

## Introduction

The prevalence of overweight and obesity has been sharply increasing in the last three decades worldwide including in China (1). The prevalence of obesity in Chinese adults was reported to increase from 3.6% in 1992 to 16.4% in 2015–19 (1). Obesity individuals are at higher risk of developing a wide range of diseases including cardiovascular disease (CVD), diabetes mellitus, chronic kidney disease, and some types of cancer (2). Nevertheless, the phenotypes of overweight and obesity are heterogeneous. Emerging evidence indicates that not all obese individuals develop metabolic disorders, and this subgroup of obese individuals is considered metabolically healthy obesity (MHO) (3). They have normal insulin sensitivity and inflammatory response and are usually free from cardiovascular metabolic risk factors (4). Similarly, the part of the normal-weight individuals with adverse metabolic status has also been reported, which is called metabolically unhealthy normal-weight (MUNW) (5).

Recent studies reveal the fact that the favorable metabolic profile might be a temporary status. A 0.5 million Chinese adults cohort showed that over one-third of the overweight or obese individuals converted from metabolic healthy to unhealthy phenotypes through 10 years of follow-up (6). Interestingly, participants with a stable MHO phenotype were found to have a comparable risk of CVD as metabolically healthy normal-weight (MHNW) individuals (7). Therefore, maintaining a healthy metabolic profile appears to be a valid approach to preventing cardiometabolic diseases (8).

The exact mechanisms underlying the metabolically healthy vs. unhealthy phenotypes remain to be explored. The dietary factors, physical activity, inflammation, and genetic factors were reported to contribute to MHO (9–12). Recent human studies suggest that adipose tissue function, body fat distribution, skeletal muscle et al. may be key factors in insulin sensitivity and metabolic phenotypes (13). Whether the effect of body composition indicators on metabolic health is consistent across all BMI categories, and whether the body fat and muscle predict metabolic phenotypes more precisely than BMI remain to be discussed.

In this present cross-sectional study, using population-based data, we aimed to determine the association between the absolute mass, distribution, and relative ratio of body fat and muscle with the metabolically unhealthy phenotypes in normal weight and overweight/obesity in Yi people in China. We also hypothesized that body fat and muscle indicators were more precise predictors than BMI in predicting metabolic phenotypes.

## Materials and methods

### Study population

The current study was based on a cross-sectional survey–The Yi Migrant Study, which was carried out in Liangshan Yi Autonomous Prefecture, Sichuan, China in 2015. The Yi Migrant Study was initiated in the 1980's and was designed to assess the cardiovascular risks as well as the determinants in Yi people using a migration epidemiology study design. Continuous field works have been conducted in different periods following the same procedure. A stratified cluster sampling method was used to recruit participants aged 20 to 80 years. Details of the sampling procedures have been described previously (14, 15).

In this migration epidemiology study, Yi people whose parents were both of Yi ethnicity can be included. Yi farmers were defined to be Yi people living in rural areas since birth. Yi migrants were defined to be Yi people who were born in rural areas and then migrated and had been living in an urban area for at least 1 year and were still living in the urban area. All participants provided written informed consent before the survey.

### Data collection

Demographic characteristics (age, sex, education level, personal annual income, et al.), disease history (hypertension, diabetes, cardiovascular disease, et al.), and health-related lifestyle factors (smoking status, drinking status, physical activity, et al.) were collected by face-to-face interviews. A standard questionnaire was administered by well-trained staff to obtain the above information (15).

Anthropometric measurements including height, weight, and body composition were taken using calibrated instruments with standard protocols. Weight and body composition were measured in light clothing by bioelectrical impedance analysis (BIA) using the body composition analyzer (BC-420, TANITA, Japan). The measurements were recorded with an accuracy of 0.1. Standing height was measured barefoot by a wall-mounted stadiometer with an accuracy of 0.1 cm.

Blood pressure was measured using a digital sphygmomanometer (Omron HEM907, Japan). For each participant, systolic blood pressure (SBP) and diastolic blood pressure (DBP) were measured three times at one-minute intervals, after at least 5 min of rest in a seated position. The average of the three measurements was recorded.

A 9-ml venous blood sample with at least 8 h of fasting overnight was collected. The samples were centrifuged, aliquoted, and immediately frozen for future tests. Fasting blood glucose (FBG, mmol/L), triglyceride (TG, mmol/L), total cholesterol (TC, mmol/L), high-density lipoprotein cholesterol (HDL-C, mmol/L), and low-density lipoprotein cholesterol (LDL-C, mmol/L) levels were tested in Beijing Hepingli Hospital.

### Definition

Body mass index (BMI) was calculated as weight in kilograms divided by the square of height in meters (kg/m^2^). In this study, BMI was categorized into normal-weight (<24 kg/m^2^) and overweight/obesity (≥24 kg/m^2^) according to the criteria for Chinese (16).

Body fat percentage (BFP, %), fat mass index (FMI, kg/m^2^), visceral fat grade (VFG), and muscle mass index (MMI, kg/m^2^) were body composition parameters of interest. BFP and VFG were directly measured by the body composition analyzer. FMI and MMI were calculated as fat mass and muscle mass in kilograms divided by the square of height in meters. The muscle/fat ratio (M/F) was calculated as fat mass divided by muscle mass.

Four cardiometabolic risk factors were used to determine metabolically unhealthy phenotypes in this study: (1) elevated blood pressure (SBP ≥ 130 mmHg and/or DBP ≥ 85 mmHg) or use of antihypertensive drugs; (2) impaired fasting glucose (IFG): FBG ≥ 5.6 mmol/L or use of medications for diabetes; (3) TG ≥ 1.7 mmol/L; (4) HDL-C <1.03 mmol/L in men or <1.30 mmol/L in women. Metabolically unhealthy was defined as meeting two or more of the cardiometabolic risk factors (17).

Combining the BMI and metabolic phenotypes, participants were divided into four categories: metabolically healthy and normal-weight (MHNW), metabolically unhealthy and normal-weight (MUNW), and metabolically healthy over-weight/obesity (MHO), and metabolically unhealthy overweight/obesity (MUO).

The definition and classification of other covariates including education, income, smoking, drinking, and physical activity were described in our previous publications (18).

### Statistical analysis

All analyses were performed using SAS statistical software (Version 9.4; SAS Institute Inc., Cary, NC, USA). A two-tailed *P*-value of < 0.05 was considered statistically significant for all analyses.

Descriptive statistics were performed stratified by the four metabolic phenotypes. Summary results were presented as mean ± standard deviation (SD) for continuous variables and number (percentage, %) for categorical variables. Differences between phenotypic categories were tested using variance analysis or the Chi-square test. The standardized prevalence of MU phenotypes was calculated based on the sex and age distributions of the 2010 China census population.

Covariance analysis was used to compare the continuous metabolic components (BMI, BP, FBG, TG, and HDL-C) and body composition parameters (BFP, VFG, FMI, MMI, and M/F) to adjust for age, sex, and residence. Data were presented as least-square mean ± standard error (SE).

To examine the association between rural-to-urban migration, age, and BMI with metabolically unhealthy (MU) phenotypes, logistics regression models were used and the following modeling strategies were applied. Model 1 included rural-to-urban migration (Yi migrants vs. Yi farmers) as the variable of interest, model 2 and model 3 included BMI and age plus model 1 in sequence and model 4 adjusted for all covariates of concern (model 3 plus sex, education, income, smoking status, drinking status, occupational physical activity, and leisure-time exercise).

To examine the linear association between body composition parameters with MU phenotypes, restricted cubic spline (RCS) functions with three knots at the 5^th^, 50^th^, and 95^th^ quantiles were fitted, in which BFP, VFG, FMI, MMI, and M/F were included as independent variables, and covariates of concern were included for adjustment. Due to the significant difference in body fat and muscle between men and women, all the analysis was performed stratified by sex. We then divided the parameters into three categories according to data distribution and integers, and use logistics regression models to assess the odds ratios (OR) and 95% confidence intervals (CI) of each parameter, with adjustment for covariates.

Finally, to evaluate whether body composition parameters perform better in predicting MU phenotypes than BMI, the receiver-operating characteristic curves (ROC) were used and the area under the curve (AUC) was calculated, and the Z-tests were used to compare the AUCs.

## Results

### Characteristics of the participants

The flowchart Supplementary Figure S1 illustrates the study sample selection. Table 1 lists the demographic and metabolic characteristics of the study participants by metabolic phenotypes. A total of 3,053 Yi people aged 20–80 years were enrolled in this study, among whom 1,884 were Yi farmers and 1,169 were rural-to-urban migrants. Of all the participants, 497 (26.31%) out of the 1,889 normal weight and 607 (52.15%) out of the 1,164 overweight/obesity were metabolically unhealthy. The age, occupational physical activity, and leisure-time exercise were significantly different between metabolically healthy and unhealthy participants both in normal weight and overweight/obesity. With the covariance analysis adjusted for age, sex, and residence, all the metabolic factors were found to be significantly different in metabolically healthy and unhealthy participants.

**Table 1:** Demographic and metabolic characteristics in participants from the Yi Migrant Study.

	**MHNW**	**MUNW**	**P**	**MHO**	**MUO**	***P***
*N* (%)	1,392 (73.69)	497 (26.31)		557 (47.85)	607 (52.15)	
Sex, *n* (%)			0.2502			0.1700
Men	459 (32.97)	178 (35.81)		177 (31.78)	216 (35.58)	
Women	933 (67.03)	319 (64.19)		380 (68.22)	391 (64.42)	
Age (years)	44.16 ± 13.71	52.72 ± 13.78	<0.0001	44.05 ± 11.97	49.92 ± 11.7	<0.0001
Age (years), n (%)			<0.0001			<0.0001
20~29	191 (13.72)	22 (4.43)		52 (9.34)	14 (2.31)	
30~39	398 (28.59)	76 (15.29)		160 (28.73)	111 (18.29)	
40~49	379 (27.23)	112 (22.54)		195 (35.01)	181 (29.82)	
50~59	192 (13.79)	104 (20.93)		77 (13.82)	160 (26.36)	
60~80	232 (16.67)	183 (36.82)		73 (13.11)	141 (23.23)	
Residence, *n* (%)			0.0005			0.0008
Farmers	1,019 (73.2)	323 (64.99)		288 (51.71)	254 (41.85)	
Rural-to-urban migrants	373 (26.8)	174 (35.01)		269 (48.29)	353 (58.15)	
Education, *n* (%)			0.8738			0.1806
Illiterate	838 (60.20)	300 (60.36)		290 (52.06)	287 (47.28)	
Primary or middle school	429 (30.82)	149 (29.98)		168 (30.16)	190 (31.30)	
High school or above	125 (8.98)	48 (9.66)		99 (17.77)	130 (21.42)	
Income (CNY/y), *n* (%)			0.9423			0.6041
<5,000	524 (37.64)	188 (37.83)		116 (20.83)	119 (19.60)	
≥5,000	868 (62.36)	309 (62.17)		441 (79.17)	488 (80.40)	
Smoking status, *n* (%)			0.0935			0.0510
Never	940 (67.53)	311 (62.58)		404 (72.53)	403 (66.39)	
Former	37 (2.66)	19 (3.82)		25 (4.49)	41 (6.76)	
Current	415 (29.81)	167 (33.60)		128 (22.98)	163 (26.85)	
Drinking status, *n* (%)			0.3919			0.0019
Never	940 (67.53)	325 (65.39)		372 (66.79)	364 (59.97)	
Former	93 (6.68)	42 (8.45)		28 (5.03)	62 (10.21)	
Current	359 (25.79)	130 (26.16)		157 (28.19)	181 (29.82)	
Occupational physical activity, *n* (%)			<0.0001			0.0028
Light	505 (36.28)	259 (52.11)		281 (50.45)	366 (60.3)	
Moderate	131 (9.41)	34 (6.84)		70 (12.57)	56 (9.23)	
Heavy	756 (54.31)	204 (41.05)		206 (36.98)	185 (30.48)	
Leisure-time exercise, *n* (%)			<0.0001			<0.0001
Light	1,163 (83.61)	369 (74.25)		397 (71.27)	338 (55.78)	
Moderate	112 (8.05)	48 (9.66)		69 (12.39)	110 (18.15)	
Heavy	116 (8.34)	80 (16.1)		91 (16.34)	158 (26.07)	
BMI (kg/m^2^, mean ± SE)^*^	20.60 ± 0.06	21.54 ± 0.09	<0.0001	26.66 ± 0.11	27.43 ± 0.12	<0.0001
SBP (mmHg, mean ± SE)^*^	113.44 ± 0.44	123.79 ± 0.69	<0.0001	120.58 ± 0.73	129.60 ± 0.75	<0.0001
DBP (mmHg, mean ± SE)^*^	69.24 ± 0.30	75.45 ± 0.47	<0.0001	73.17 ± 0.46	79.44 ±0.47	<0.0001
FBG (mmol/L, mean ± SE)^*^	5.15 ± 0.04	6.09 ± 0.06	<0.0001	5.16 ± 0.10	6.16 ± 0.09	<0.0001
TG (mmol/L, mean ± SE)^*^	1.07 ± 0.02	1.87 ± 0.03	<0.0001	1.14 ± 0.05	2.19 ± 0.04	<0.0001
HDL (mmol/L mean ± SE)^*^	1.33 ± 0.01	1.07 ± 0.01	<0.0001	1.27 ± 0.01	1.03 ± 0.01	<0.0001

MHNW, metabolically healthy and normal-weight; MUNW, metabolically unhealthy and normal-weight; MHO, metabolically healthy overweight/obesity; MUO, metabolically unhealthy overweight/obesity; BMI, body mass index; SBP, systolic blood pressure; DBP, diastolic blood pressure; FBG, fasting blood glucose; TG triglyceride; HDL, high-density lipoprotein; SE, standard error.

* Covariance analysis adjusted for age, sex, and residence.

### Prevalence of metabolically unhealthy phenotypes in Yi people

Stratified by BMI, the crude prevalence of MUNW and MUO in Yi farmers was significantly lower than in Yi migrants. After the adjustment for age and sex, the standardized prevalence of MUNW in Yi farmers and Yi migrants was still significantly different, while the standardized prevalence of MUO in the two groups was no different (see Supplementary Figures S2, S3).

**Figure 1:**
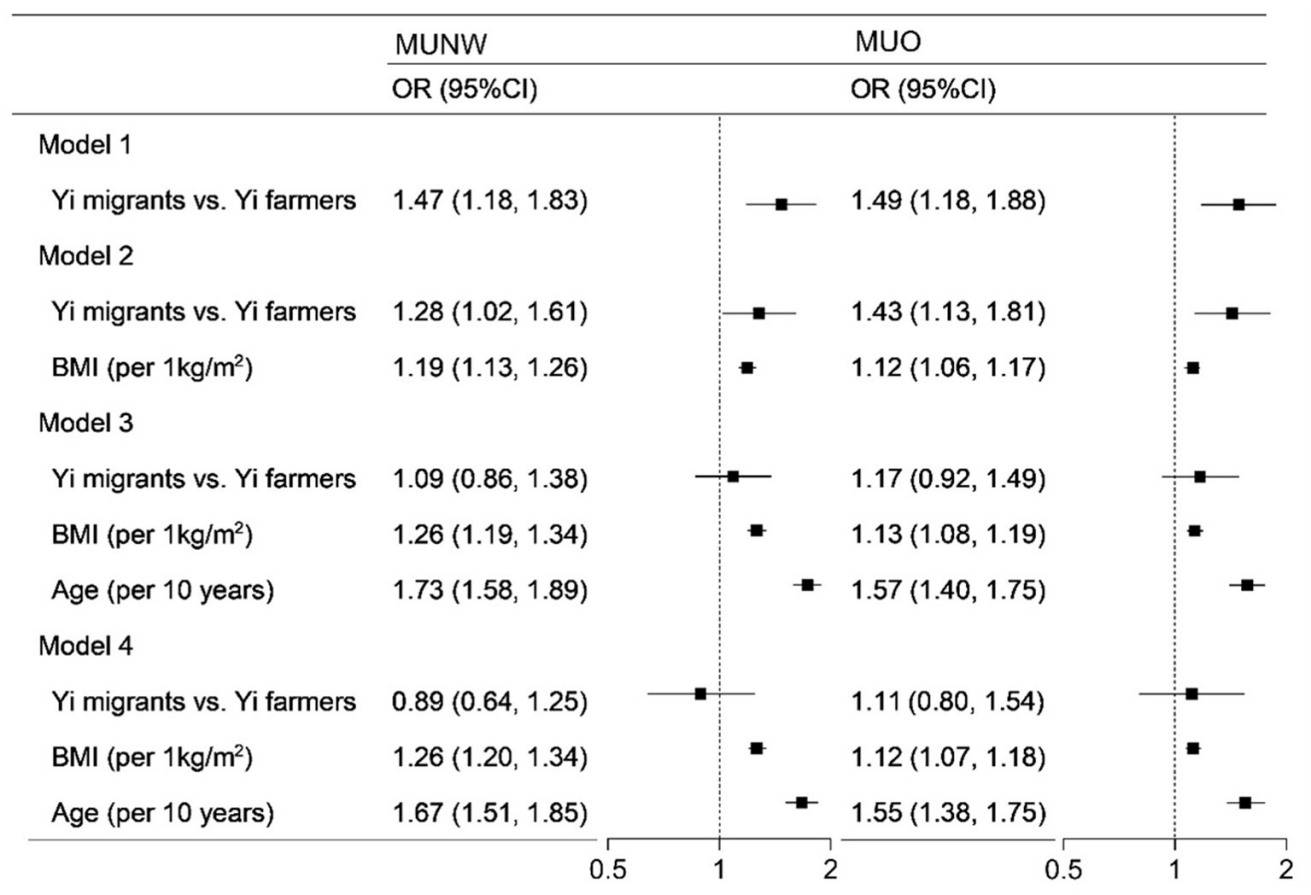
Logistics regression analysis of the association between rural-to-urban migration, age, and BMI with metabolically unhealthy phenotype. MUNW, metabolically unhealthy and normal-weight; MUO, metabolically unhealthy overweight/obesity; OR, odds ratio; BMI, body mass index. In the MUNW models, the MHNW was the control, and in the MUO models, the MHO was the control. Model 1 included rural-to-urban migration. Model 2 included model 1 plus BMI. Model 3 included model 2 plus BMI and age. Model 4 included model 3 plus sex, education, income, smoking status, drinking status, occupational physical activity, and leisure-time exercise.

The results of logistics regression models in Figure 1 show the association between rural-to-urban migration, BMI, and age with metabolically unhealthy phenotypes in Yi people. Without adjustment, Yi migrants were at nearly 1.5-fold higher odds of MU than Yi farmers. With the sequential adjustment for BMI and age, the ORs of rural-to-urban migration gradually decreased and were no more significant. With the full adjustment for demographic characteristics and health-related lifestyle factors, the OR of MU was 1.26 per 1 kg/m^2^ of BMI and 1.67 per 10 years of age in normal weight. In overweight/obesity, the ORs were 1.12 and 1.55 for BMI and age, respectively.

### Association between body composition with metabolically unhealthy phenotypes

Covariance analysis of body composition between metabolically healthy and unhealthy phenotypes was shown in Table 2. After adjustment for age and residence, the BFP, FMI, VFG, and MMI in metabolically healthy participants were significantly lower than in those metabolically unhealthy both in men and women. The MMI in overweight/obese men was an exception, the difference between the phenotypic groups was not significant. The M/F was found to be significantly higher in MHNW and MHO than in MUNW and MUO.

**Table 2:** Covariance analysis of body composition between metabolically healthy and unhealthy participants by sex and BMI.

	**BMI**<**24 kg/m**^**2**^			**BMI** ≥**24 kg/m**^**2**^		
	**MHNW**	**MUNW**	**P**	**MHO**	**MUO**	***P***
**Men**						
BFP (%)	17.08 ± 0.22	19.24 ± 0.32	<0.0001	25.69 ± 0.22	26.67 ± 0.21	0.0007
FMI (kg/m^2^)	3.59 ± 0.06	4.21 ± 0.09	<0.0001	6.92 ± 0.11	7.33 ± 0.11	0.0043
VFG	6.81 ± 0.17	8.48 ± 0.25	<0.0001	13.19 ± 0.14	13.73 ± 0.14	0.0035
MMI (kg/m^2^)	16.12 ± 0.05	16.47 ± 0.08	<0.0001	18.74 ± 0.08	18.87 ± 0.07	0.1644
M/F	4.80 ± 0.07	4.15 ± 0.11	<0.0001	2.80 ± 0.03	2.65 ± 0.03	0.0003
**Women**						
BFP (%)	28.28 ± 0.16	30.50 ± 0.25	<0.0001	37.94 ± 0.18	39.03 ± 0.20	<0.0001
FMI (kg/m^2^)	5.91 ± 0.05	6.63 ± 0.08	<0.0001	10.19 ± 0.11	10.85 ± 0.12	<0.0001
VFG	4.09 ± 0.05	4.81 ± 0.09	<0.0001	7.70 ± 0.08	8.16 ± 0.08	<0.0001
MMI (kg/m^2^)	13.88 ± 0.03	14.09 ± 0.04	<0.0001	15.52 ± 0.03	15.71 ± 0.04	<0.0001
M/F	2.48 ± 0.02	2.18 ± 0.04	<0.0001	1.56 ± 0.01	1.49 ± 0.01	<0.0001

MHNW, metabolically healthy and normal-weight; MUNW, metabolically unhealthy and normal-weight; MHO, metabolically healthy overweight/obesity; MUO, metabolically unhealthy overweight/obesity; BMI, body mass index; BFP, body fat percentage; FMI, fat mass index; VFG, visceral fat grade; MMI, muscle mass index; M/F, muscle-to-fat ratio. Covariance analysis adjusted for age and residence. Data were shown as mean ± SE (standard error).

**Figure 2:**
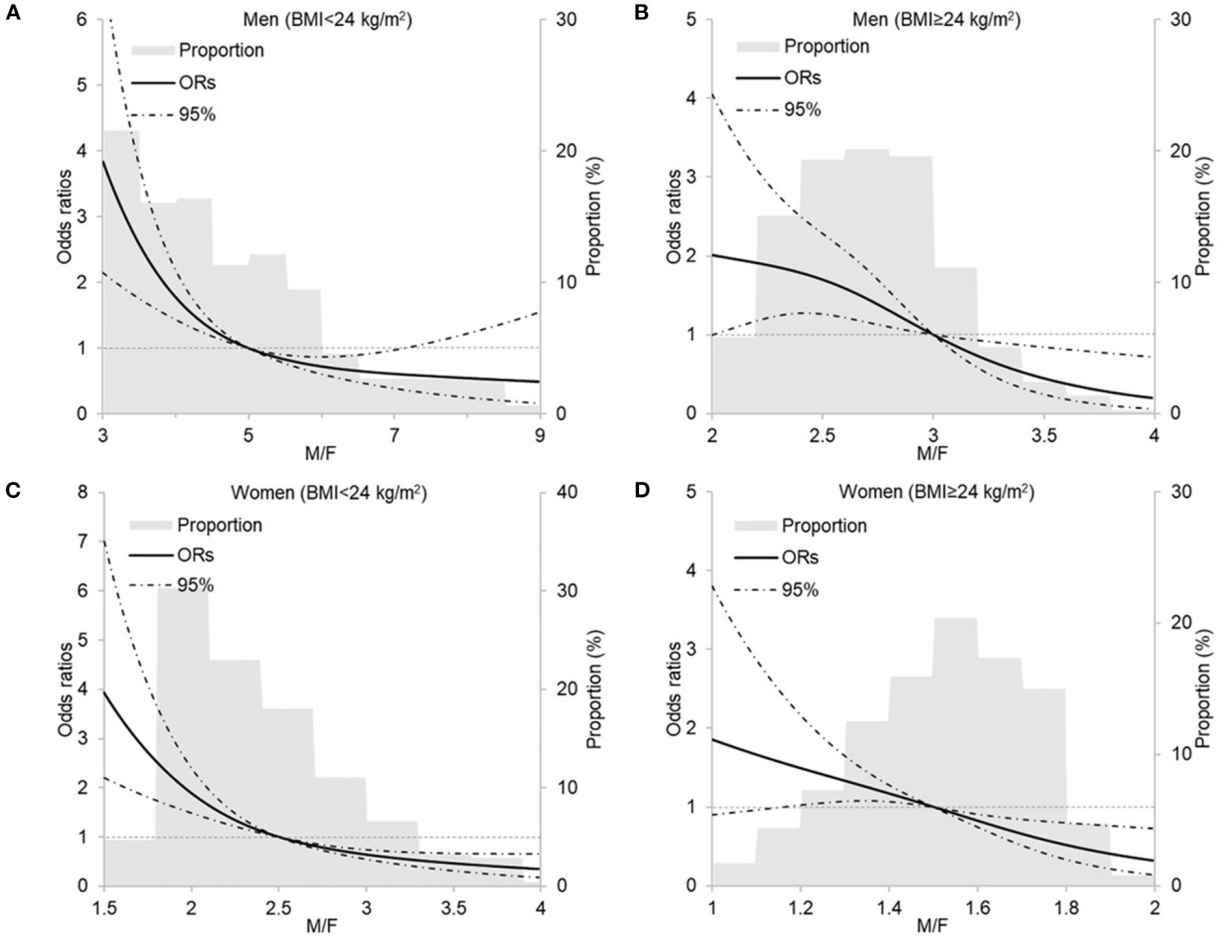
Restricted cubic spline analysis of the relationship between M/F with metabolically unhealthy phenotype by sex and BMI. **(A)** men, BMI < 24 kg/m^2^, P _linear_ < 0.0001; **(B)** men, BMI ≥ 24 kg/m^2^, P _linear_ = 0.7122; **(C)** women, BMI <24 kg/m^2^, P _linear_ < 0.0001; **(D)** women, BMI ≥ 24 kg/m^2^, P _linear_ = 0.2234. M/F, muscle-to-fat ratio; BMI, body mass index. Models included age, residence, education, income, smoking status, drinking status, occupational physical activity, and leisure-time exercise.

The RCS analysis shows the linear relationship between body composition parameters and MU phenotypes by sex and BMI (see Figure 2 and Supplementary Figures S4–S7). With the adjustment for demographic characteristics and health-related lifestyle factors, BFP, FMI, VFG, and MMI show positive relationships with MHNW and MUO both in men and women. Figure 2 shows that with the increase in muscle/fat ratio, the ORs of MUNW and MUO descend.

**Figure 3:**
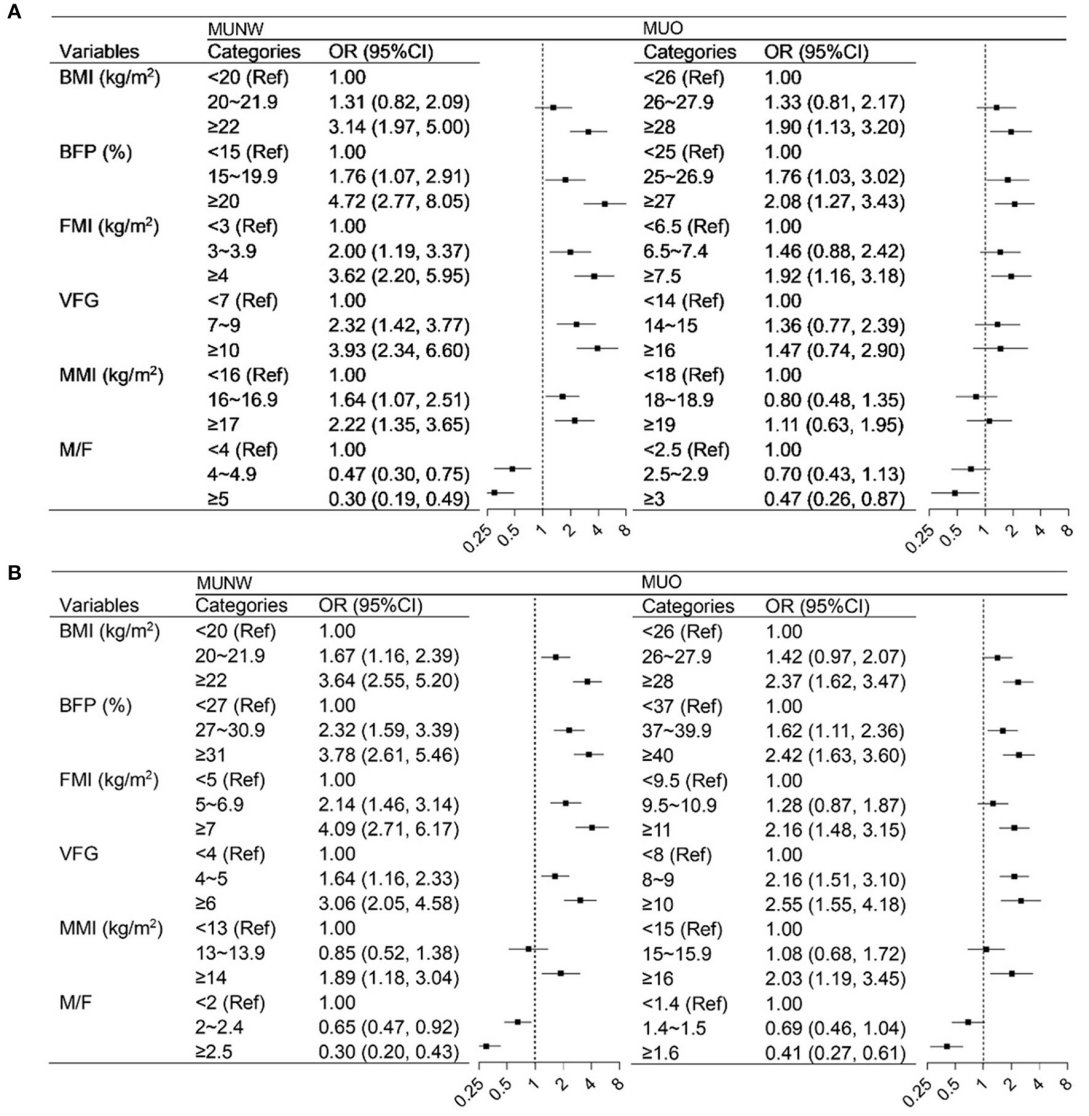
Logistics regression analysis of the association between body composition with metabolically unhealthy phenotype by sex and BMI. **(A)** men, **(B)** women. BMI, body mass index; BFP, body fat percentage; FMI, fat mass index; VFG, visceral fat grade; MMI, muscle mass index; M/F, muscle-to-fat ratio. In the MUNW models, the MHNW was the control, and in the MUO models, the MHO was the control. Models were adjusted for age, residence, education, income, smoking status, drinking status, occupational physical activity, and leisure-time exercise.

The continuous body fat and muscle parameters were divided into three categories according to tertiles and their association with MU phenotypes was assessed using logistic regression models by sex (see Figure 3). Each of the parameters was evaluated in a multivariable model separately, with the adjustment for demographic characteristics and health-related lifestyle factors. In men participants, the higher BFP and FMI categories were positively associated with MUNW and MUO. VFG and MMI were associated with higher odds of MUNW but not MUO. The higher M/F categories were associated with decreased odds of both MUNW and MUO. In women, all these parameters were significantly associated with MU phenotypes. The association between BFP, FMI, VFG, and MMI was positive while the association of M/F was negative. As the results show the consistent association between body fat and muscle with MU across the BMI categories, we then evaluated the association in the whole population. Supplementary Table S1 shows the strong association between BFP, FMI, VFG, MMI, and M/F with MU phenotype both in men and women.

**Figure 4:**
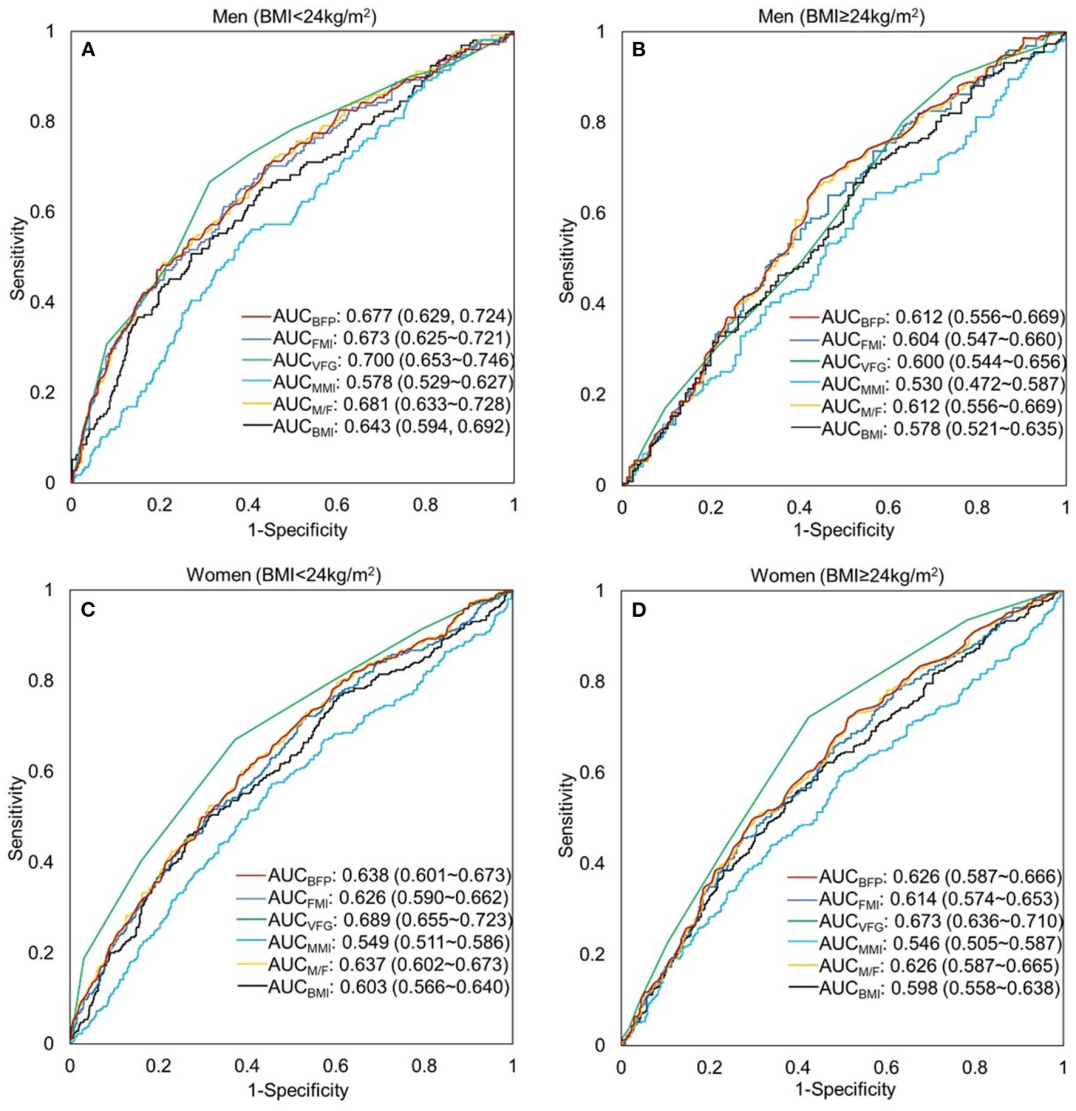
Receiver-operating characteristic (ROC) curve of the body composition indicators to predict metabolically unhealthy phenotype by sex and BMI. **(A)** men, BMI < 24 kg/m^2^; **(B)** men, BMI ≥ 24 kg/m^2^; **(C)** women, BMI <24 kg/m^2^; **(D)** women, BMI≥24 kg/m^2^. BFP, body fat percentage (%); FMI, fat mass index (kg/m^2^); VFG, visceral fat grade; MMI, muscle mass index (kg/m^2^); M/F, muscle-to-fat ratio; BMI, body mass index (kg/m^2^).

The ROCs of the body fat and muscle for predicting MU phenotypes by sex and BMI are shown in Figure 4. These parameters did not show a favorable predicting value of MUNW and MUO in men and women (~0.6–0.7). But they have better performance in prediction than BMI except for MMI (*P* < 0.05 for all comparisons). A better performance was found in normal weight than that in overweight/obesity.

## Discussion

In this cross-section study, we demonstrated the prevalence of metabolically unhealthy phenotypes and evaluated the association of absolute mass (BFP, FMI, MMI), distribution (VFG), and relative ratio (M/F) of body fat and muscle with the MU phenotypes in normal weight and overweight/obesity in Yi people. Our results verified the consistent effects of body fat and muscle mass on metabolic health across the normal-weight and overweight/obesity categories, and also illustrated the better performance of body composition indicators than BMI in predicting MU phenotypes.

The idea and the clinical implication of MHO have drawn much discussion in the past few years (19). The research on MHO is important because the existence of a metabolic healthy phenotype among obesity might provide an effective way for obesity management and treatment. Due to the lack of standard criteria to define metabolically healthy obesity, the prevalence of MHO can be greatly varied (20, 21). In this study, we assessed that approximately half of the overweight/obese adults in Yi people were metabolically healthy. We also found that although normal-weight, 1/4 to 1/3 of participants had unhealthy metabolic phenotypes. The proportions differed by residence and increased by age and BMI. Even though this study and our previous studies showed that rural-to-urban migrants were at higher risk of metabolic disorders (22–24), the variance could be partly attributed to demographic and increased body weight. The results showed that the disparities between Yi farmers and rural-to-urban migrants were no more significant after adjusting for age and BMI, which indicated the essential role of age and body weight increase on metabolic unhealthy phenotypes.

Along with genetic and lifestyle factors (25, 26), BMI and total adiposity are positively correlated with cardiometabolic disease risk at the population level (27). BMI is a widely used parameter for evaluating obesity, while BMI is unable to distinguish the relative mass of body fat and lean mass, nor estimate the body fat distribution. In this study, using the bioelectrical impedance analysis method, we assessed the mass (BFP and FMI) and distribution (VFG) of body fat. The results indicated that no matter the BMI categories and sex, both mass and distribution of body fat showed a positive relationship with metabolically unhealthy phenotypes. A recent study in China showed coincident results of the relationships (28). Lv et al. (28) measured the body composition indices by quantitative computed tomography and found that total adipose tissue (TAT), visceral adipose tissue (VAT), and VAT/TAT were positively associated with a higher risk of MU phenotypes across BMI categories. In our study, we did not assess the subcutaneous fat, which showed a significant difference between metabolically healthy and unhealthy participants (28, 29). Another parameter of fat distribution in this study, VFG, showed a strong association with metabolic health and showed better predicting performance than the other indicators. The effect of visceral fat has also been proved in previous studies (30, 31).

Muscle mass was regarded as a protective factor against MU phenotypes in previous studies (32, 33). However, in this study, the highest category of muscle mass index was at increased risk of being metabolically unhealthy. It might be due to the concomitance of muscle and fat mass at the individual level. Kim et al. (32) compared the muscle mass and quality between metabolically healthy and unhealthy phenotypes and concluded that not only muscle mass but also muscle quality are associated with metabolic health. In the present study, we calculated the muscle-to-fat ratio to assess the effect of the relative ratio of muscle and fat on metabolic health. The results showed that increased M/F values were beneficial to a healthier metabolic phenotype. The fat-to-muscle ratio has been introduced as a new anthropometric indicator by several researchers (34–36). However, we regard the muscle-to-fat ratio as a more significant indicator because it provided the approach to reducing metabolic disorder risks by elevating the muscle mass and simultaneously reducing the fat mass. A previous study also suggested that the muscle-to-fat ratio was a better indicator than the fat-to-muscle ratio in quantifying insulin resistance (37).

Our study has a few importance for clinical and public health implications. The study verified the heterogeneity of obesity, and also reminded the implicit cardiometabolic disease risk in the normal-weight. We emphasized the importance of body weight management by monitoring BMI at the population level. Despite the weakness of distinguishing body composition, BMI is an intuitive and economical approach for indicating the exceeded body adipose tissues. The study confirmed the linear positive association between body fat (BFP, FMI, and VFG) with MU phenotypes, and also proved a better performance in predicting MU phenotypes than BMI. The results called for the utilization of body composition measurement for a more precise obesity evaluation and prevention when the health resources were accessible. Additionally, since 1/4 to 1/3 of the normal-weight participants were metabolically unhealthy, it is essential to maintain a healthy lifestyle and monitor blood pressure, glucose, and lipid metabolism at an appropriate interval in this cardiometabolic low-risk population.

One of the limitations of our study lies in the cross-sectional nature of the study design. The statistical association can be found, but, no causal inference can be reliably established. Secondly, the measurements of the body fat and muscle were dependent on the bioelectrical impedance analysis method, rather than the more accurate dual-energy X-rays absorptiometry (DXA). While the BIA has the advantage of safety, cost, and portability over DXA in the large-scale population-based field survey. And fortunately, BIA shows satisfying agreement with DXA in the real-world setting (38). We also acknowledge that AUCs around 0.6 in our study were not good enough to distinguish MUNW and MHO. More precise indicators were pending to be discovered in the future. Finally, we did not collect data on diet, which is a major contributor to metabolic phenotypes. The lack of dietary data limits the detection of risk factors and the essential adjustment in the multivariable models. Further prospective studies are needed to collect comprehensive data and verify the relationship between body fat and muscle with metabolic trajectories.

## Conclusions

In this cross-sectional study, using the population-based data, we observed that the metabolically unhealthy phenotypes were prevalent both in normal-weight and overweight/obesity in Yi people. The results showed the positive association of BFP, FMI, VFG, and the negative association of M/F with MU phenotypes across the BMI categories in Yi people. Measurement of body fat and muscle could provide a more precise approach for the management and prevention of obesity-related cardiometabolic risks.

## Data availability statement

The original contributions presented in the study are included in the article/Supplementary material, further inquiries can be directed to the corresponding author.

## Ethics statement

The studies involving human participants were reviewed and approved by bio Ethical Committee of Institute of Basic Medical Sciences, Chinese Academy of Medical Sciences. The patients/participants provided their written informed consent to participate in this study.

## Author contributions

GS designed the study and supervised data collection. YW analyzed the data, interpreted results, and drafted the manuscript. LP, SW, WY, FY, ZL, and ZY participated in data collection. All authors have approved the submitted versions.

## Funding

This work was supported by grants from National Natural Science Foundation of China (No. 81273158).

## Conflict of interest

The authors declare that the research was conducted in the absence of any commercial or financial relationships that could be construed as a potential conflict of interest.

## Publisher's note

All claims expressed in this article are solely those of the authors and do not necessarily represent those of their affiliated organizations, or those of the publisher, the editors and the reviewers. Any product that may be evaluated in this article, or claim that may be made by its manufacturer, is not guaranteed or endorsed by the publisher.
