# Supplementary material for: Body fat and muscle were associated with metabolically unhealthy phenotypes in normal weight and overweight/obesity in Yi people: A cross-sectional study in Southwest China
Source: Front Public Health. 2022 Oct 6;10:1020457. doi: 10.3389/fpubh.2022.1020457 (PMC9582532; doi:10.3389/fpubh.2022.1020457)
Supplement: Supplementary file 1 [file Table_1.DOCX]

| 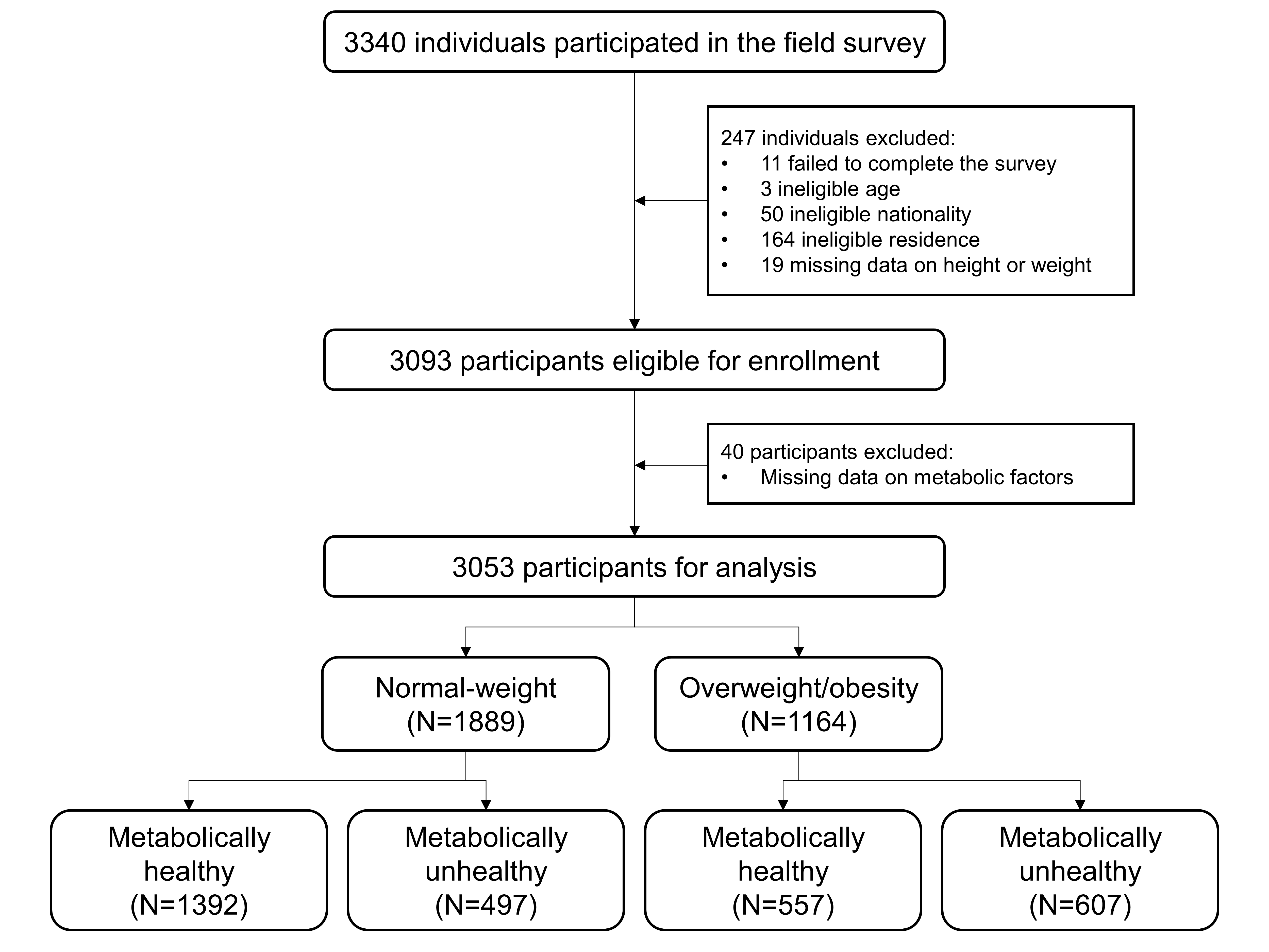 |
| --- |
| **Figure S1.** Flowchart of the study sample selection |


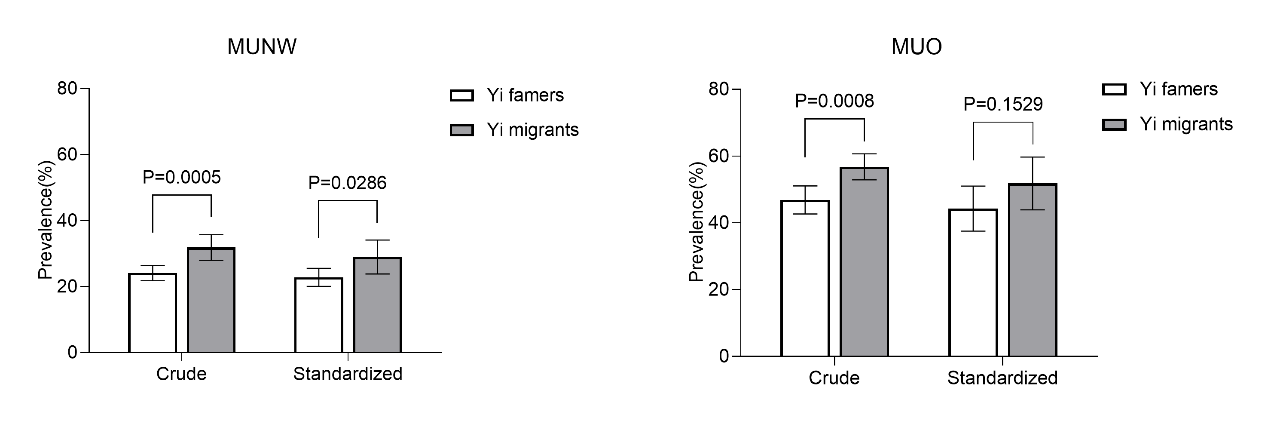


**Figure S2.** The crude and standardized prevalence of MUNW in normal-weight and MUO in overweight/obesity in Yi farmers and Yi migrants. MUNW: metabolically unhealthy and normal-weight; MUO: metabolically unhealthy overweight/obesity.

| (a) | 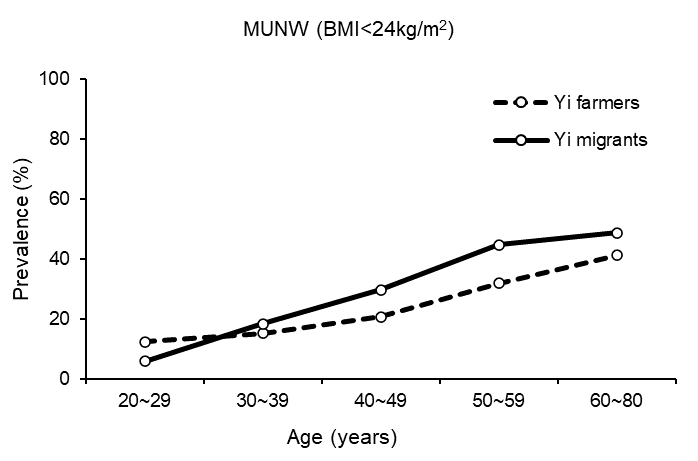 | (b) | 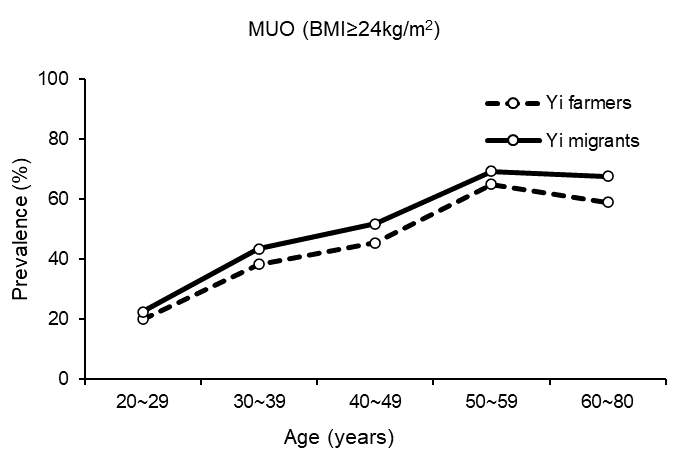 |
| --- | --- | --- | --- |
| (c) | 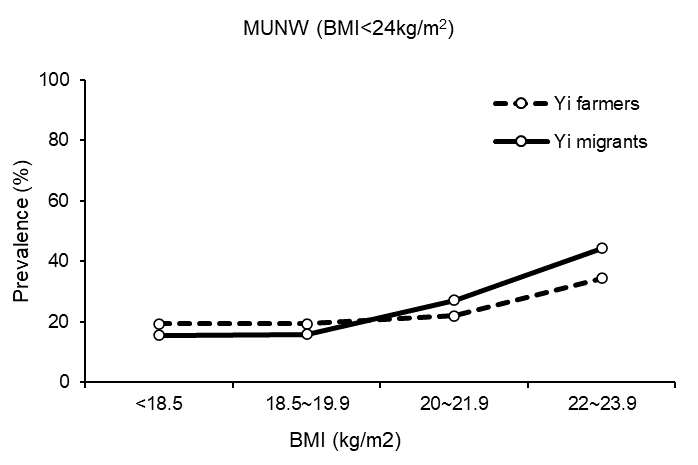 | (d) | 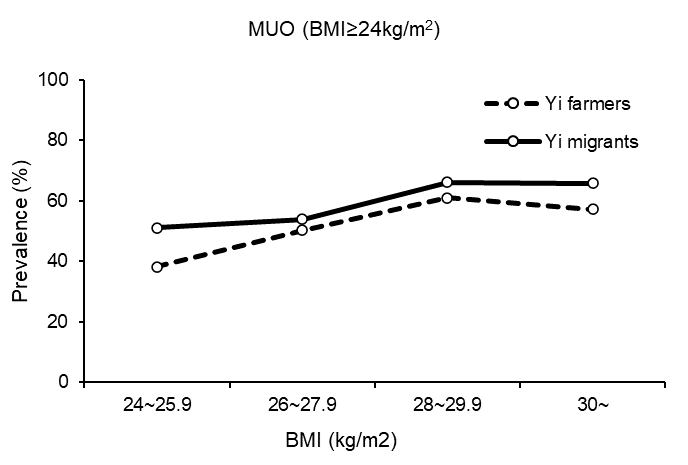 |

**Figure S3.** Trends of metabolically unhealthy phenotype by age and BMI in Yi farmers and Yi migrants. MUNW: metabolically unhealthy and normal-weight; MUO: metabolically unhealthy overweight/obesity. (a) prevalence of MUNW in normal-weight by age; (b) prevalence of MUO in overweight/obesity by age; (c) prevalence of MUNW in normal-weight by BMI; (d) prevalence of MUO in overweight/obesity by BMI.


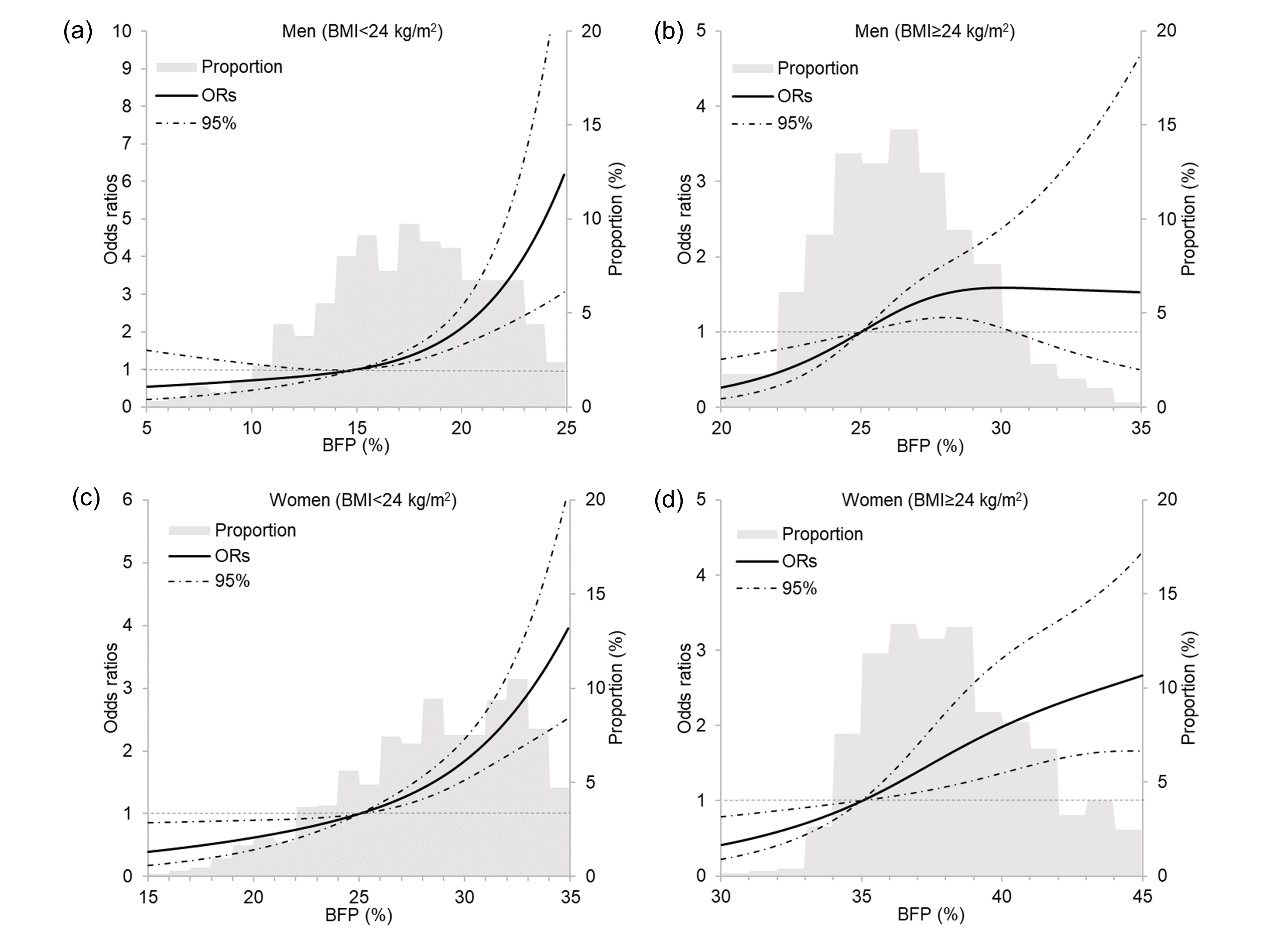


**Figure S4.** Restricted cubic spline analysis of the relationship between BFP with metabolically unhealthy phenotype by sex and BMI. (a) men, BMI<24kg/m^2^, P _linear_=0.3260; (b) men, BMI≥24 kg/m^2^, P _linear_=0.0037; (c) women, BMI<24kg/m^2^, P _linear_=0.0293; (d) women, BMI≥24 kg/m^2^, P _linear_=0.0070. BFP: body fat percentage; BMI: body mass index. Models included age, residence, education, income, smoking status, drinking status, occupational physical activity, and leisure-time exercise.


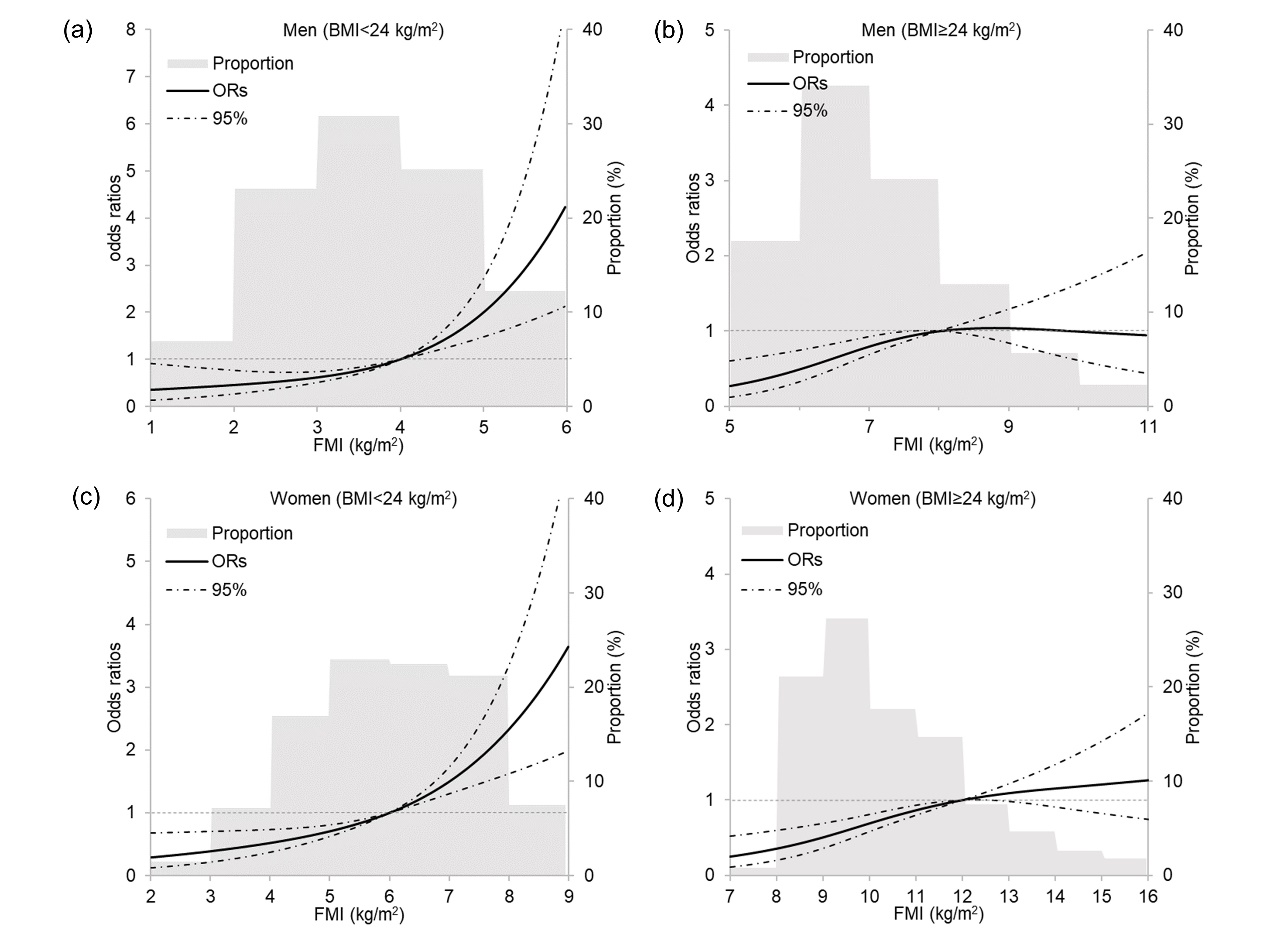


**Figure S5.** Restricted cubic spline analysis of the relationship between FMI with metabolically unhealthy phenotype by sex and BMI. (a) men, BMI<24kg/m^2^, P _linear_=0.2574; (b) men, BMI≥24 kg/m^2^, P _linear_=0.0038; (c) women, BMI<24kg/m^2^, P _linear_=0.0257; (d) women, BMI≥24 kg/m^2^, P _linear_=0.0021. FMI: fat mass index; BMI: body mass index. Models included age, residence, education, income, smoking status, drinking status, occupational physical activity, and leisure-time exercise.


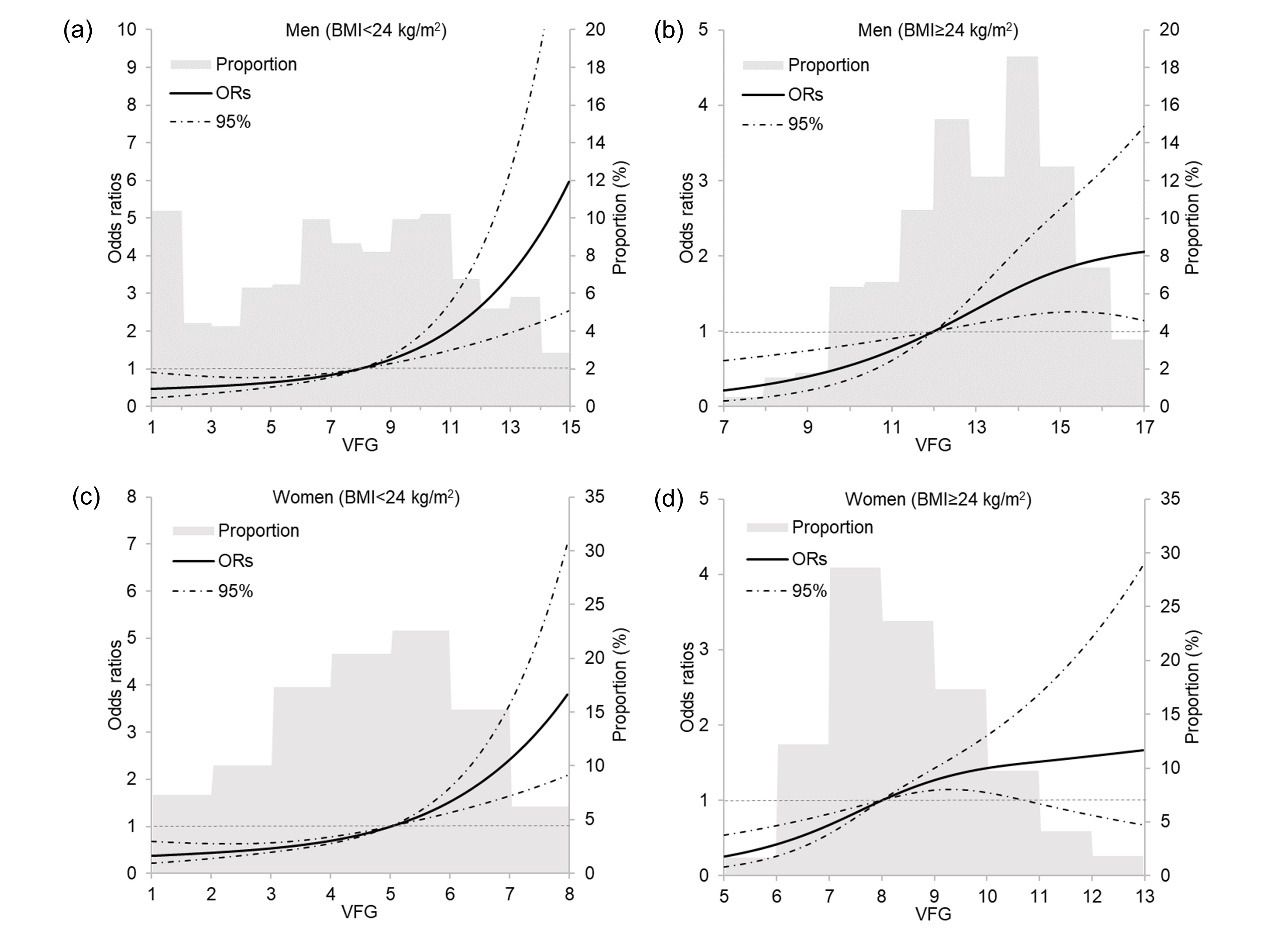


**Figure S6.** Restricted cubic spline analysis of the relationship between VFG with metabolically unhealthy phenotype by sex and BMI. (a) men, BMI<24kg/m^2^, P _linear_=0.3935; (b) men, BMI≥24 kg/m^2^, P _linear_=0.0046; (c) women, BMI<24kg/m^2^, P _linear_=0.2481; (d) women, BMI≥24 kg/m^2^, P _linear_=0.0007. VFG: visceral fat grade; BMI: body mass index. Models included age, residence, education, income, smoking status, drinking status, occupational physical activity, and leisure-time exercise.


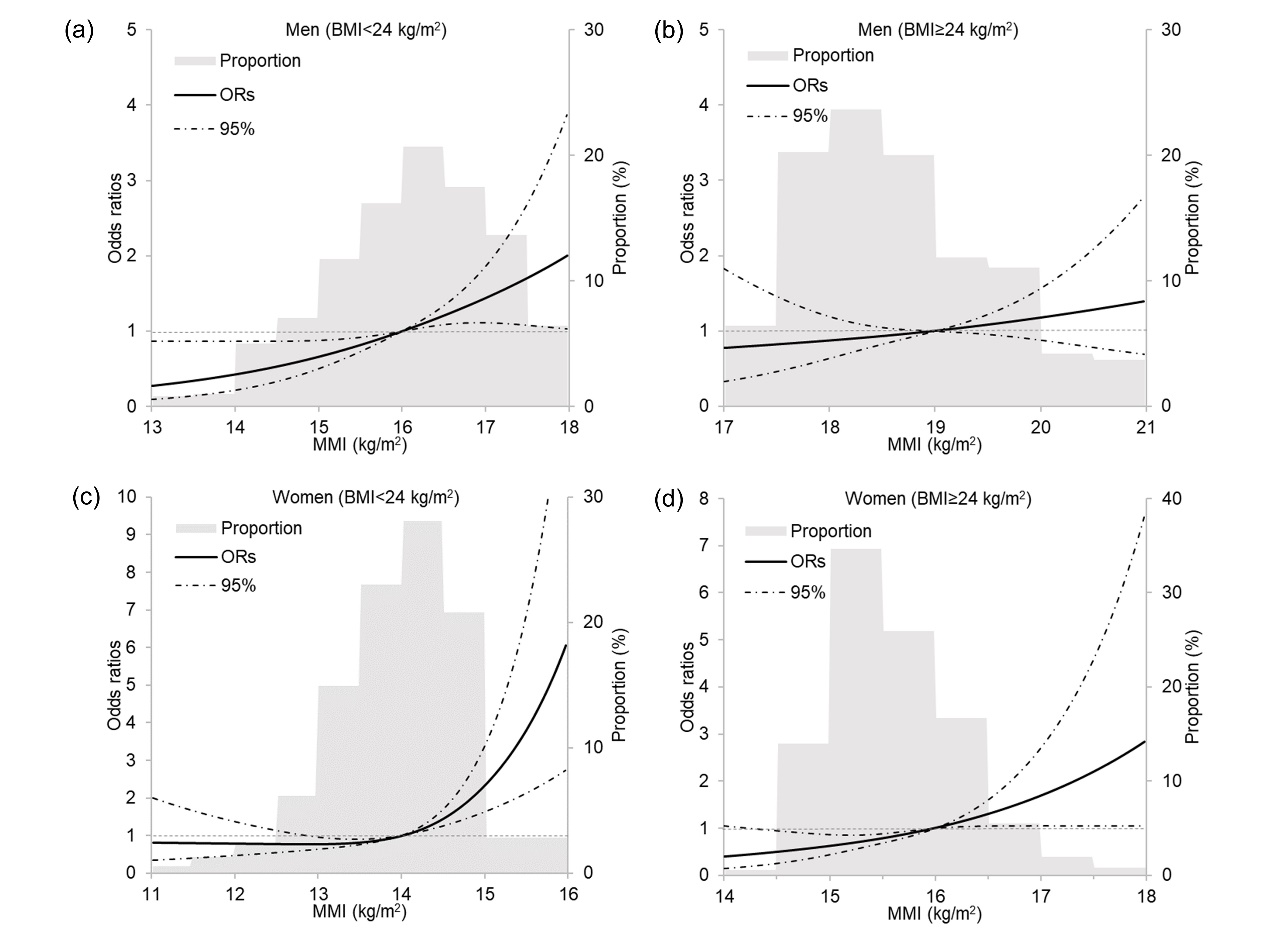


**Figure S7.** Restricted cubic spline analysis of the relationship between MMI with metabolically unhealthy phenotype by sex and BMI. (a) men, BMI<24kg/m^2^, P _linear_=0.0521; (b) men, BMI≥24 kg/m^2^, P _linear_=0.6850; (c) women, BMI<24kg/m^2^, P _linear_=0.8618; (d) women, BMI≥24 kg/m^2^, P _linear_=0.1858. MMI: muscle mass index; BMI: body mass index. Models included age, residence, education, income, smoking status, drinking status, occupational physical activity, and leisure-time exercise.

**Table S1.** Logistics regression analysis of the association between body composition with metabolically unhealthy phenotype by sex.

|  | Men |  | Women |  |
| --- | --- | --- | --- | --- |
| Variables | Categories | OR (95%CI) | Categories | OR (95%CI) |
| BMI (kg/m^2^) | <20 (Ref) | 1.00 | <20 (Ref) | 1.00 |
|  | 20~23.9 | 1.98 (1.33~2.93) | 20~23.9 | 2.49 (1.83~3.39) |
|  | 24~27.9 | 4.01 (2.62~6.15) | 24~27.9 | 4.60 (3.32~6.37) |
|  | ≥28 | 7.10 (4.10~12.29) | ≥28 | 9.38 (6.30~13.97) |
| BFP (%) | <15 (Ref) | 1.00 | <27 (Ref) | 1.00 |
|  | 15~19.9 | 1.75 (1.07~2.88) | 27~31.9 | 2.43 (1.70~3.46) |
|  | 20~24.9 | 4.49 (2.77~7.28) | 32~36.9 | 4.20 (2.98~5.94) |
|  | ≥25 | 8.45 (5.10~14.00) | ≥37 | 8.66 (6.06~12.39) |
| FMI (kg/m^2^) | <3 (Ref) | 1.00 | <5 (Ref) | 1.00 |
|  | 3~4.9 | 2.20 (1.39~3.50) | 5~6.9 | 2.09 (1.43~3.07) |
|  | 5~6.9 | 5.72 (3.54~9.24) | 7~8.9 | 4.18 (2.85~6.11) |
|  | ≥7 | 9.37 (5.54~15.84) | ≥9 | 7.62 (5.22~11.12) |
| VFG | <7 (Ref) | 1.00 | <15 (Ref) | 1.00 |
|  | 7~9.9 | 2.55 (1.59~4.09) | 15~19.9 | 1.63 (1.15~2.30) |
|  | 10~12.9 | 3.83 (2.45~5.99) | 20~24.9 | 3.16 (2.26~4.41) |
|  | ≥13 | 8.17 (5.18~12.89) | ≥25 | 7.18 (5.03~10.26) |
| MMI (kg/m^2^) | <16 (Ref) | 1.00 | <14 (Ref) | 1.00 |
|  | 16~16.9 | 1.65 (1.09~2.50) | 14~14.9 | 2.35 (1.78~3.09) |
|  | 17~17.9 | 2.71 (1.80~4.10) | 15~15.9 | 3.89 (2.90~5.23) |
|  | ≥18 | 4.00 (2.68~5.97) | ≥16 | 7.52 (5.10~11.08) |
| M/F | <3 (Ref) | 1.00 | <1.5 (Ref) | 1.00 |
|  | 3~3.9 | 0.57 (0.41~0.80) | 1.5~1.9 | 0.49 (0.37~0.65) |
|  | 4~4.9 | 0.26 (0.17~0.41) | 2~2.4 | 0.26 (0.19~0.36) |
|  | ≥5 | 0.16 (0.10~0.26) | ≥2.5 | 0.12 (0.08~0.17) |

BMI: body mass index; BFP: body fat percentage; FMI: fat mass index; VFG: visceral fat grade; MMI: muscle mass index; M/F: muscle-to-fat ratio. Models were adjusted for age, residence, education, income, smoking status, drinking status, occupational physical activity, and leisure-time exercise.
